# Distinct Fractions of an *Artemisia scoparia* Extract Contain Compounds With Novel Adipogenic Bioactivity

**DOI:** 10.3389/fnut.2019.00018

**Published:** 2019-03-08

**Authors:** Anik Boudreau, Alexander Poulev, David M. Ribnicky, Ilya Raskin, Thirumurugan Rathinasabapathy, Allison J. Richard, Jacqueline M. Stephens

**Affiliations:** ^1^Pennington Biomedical Research Center, Baton Rouge, LA, United States; ^2^Department of Plant Biology and Pathology, Rutgers University, New Brunswick, NJ, United States; ^3^Department of Biological Sciences, Louisiana State University, Baton Rouge, LA, United States

**Keywords:** adipocyte, plants-medicinal, botanical extract, adipogenesis, fat cell, activity-guided fractionation, 3T3-L1 adipocyte

## Abstract

Adipocytes are important players in metabolic health and disease, and disruption of adipocyte development or function contributes to metabolic dysregulation. Hence, adipocytes are significant targets for therapeutic intervention in obesity and metabolic syndrome. Plants have long been sources for bioactive compounds and drugs. In previous studies, we screened botanical extracts for effects on adipogenesis *in vitro* and discovered that an ethanolic extract of *Artemisia scoparia* (SCO) could promote adipocyte differentiation. To follow up on these studies, we have used various separation methods to identify the compound(s) responsible for SCO's adipogenic properties. Fractions and subfractions of SCO were tested for effects on lipid accumulation and adipogenic gene expression in differentiating 3T3-L1 adipocytes. Fractions were also analyzed by Ultra Performance Liquid Chromatography- Mass Spectrometry (UPLC-MS), and resulting peaks were putatively identified through high resolution, high mass accuracy mass spectrometry, literature data, and available natural products databases. The inactive fractions contained mostly quercetin derivatives and chlorogenates, including chlorogenic acid and 3,5-dicaffeoylquinic acid, which had no effects on adipogenesis when tested individually, thus ruling them out as pro-adipogenic bioactives in SCO. Based on these studies we have putatively identified the principal constituents in SCO fractions and subfractions that promoted adipocyte development and fat cell gene expression as prenylated coumaric acids, coumarin monoterpene ethers, 6-demethoxycapillarisin and two polymethoxyflavones.

## Introduction

Obesity, one of the great public health challenges of our time, is a major risk factor for type 2 diabetes mellitus (T2DM) and cardiovascular disease (1, 2). Inhibition of adipocyte differentiation was once viewed as a promising therapeutic strategy for preventing or treating obesity and its metabolic consequences. However, it is now known that obese and insulin resistant states are associated with impaired adipogenesis and that limiting adipose tissue expansion leads to ectopic lipid deposition and metabolic dysregulation (3–6). Thiazolidinediones (TZDs) are a class of antidiabetic drugs that both enhance adipogenesis and improve whole-body insulin sensitivity in the presence of increased adipose tissue mass (7). Clinical use of TZDs has declined substantially since 2007, in response to concerns over safety and side effects that may not occur from botanical alternatives of TZDs. Nevertheless, adipocyte differentiation continues to be a therapeutic target of interest for intervention in metabolic related diseases including obesity and T2DM.

Botanicals have a long history of medicinal use in many cultures, and countless drugs have been derived from plants, including metformin, a compound derived from French lilac that was used in traditional herbal medicine for centuries to lower blood glucose (8, 9). Currently, metformin is the preferred first line treatment and most commonly prescribed medication for T2DM. Since plant extracts represent a potential source of novel therapeutic compounds, we have performed screening experiments to identify botanical extracts that could enhance adipocyte development. We identified an extract of *Artemisia scoparia* (SCO) as having positive effects on adipocyte differentiation and endocrine function both *in vitro* and *in vivo* (10, 11). We have also described the inhibition of lipolysis, both *in vitro* and *in vivo*, as an additional mechanism involved in SCO's metabolically beneficial effects (12). The focus of the current study was to identify the constituents of SCO responsible for its adipogenic effects.

Initially, three crude partitions of the SCO parent extract were generated with water, hexane, and ethyl acetate (W, H, and EA). Each of these crude fractions was tested for the ability to promote adipogenesis in 3T3-L1 cells by assessing both lipid accumulation and adipocyte marker gene expression. Next, we performed successive rounds of fractionation and testing to separate active from inactive fractions. Ultra Performance Liquid Chromatography- Mass Spectrometry (UPLC-MS) analysis was performed on all fractions, and compounds were putatively identified using high resolution, high mass accuracy mass spectrometry, literature data, and available databases. We determined that the predominant constituents identified in the inactive fractions were chlorogenates and quercetin derivatives, while the active fractions primarily contained prenylated coumaric acids (PCAs), coumarin monoterpene ethers, 6-demethoxycapillarisin, as well as two polymethoxyflavones. In addition, we observed that some fractions with no common compounds were active in our adipogenesis assay, suggesting that more than one component in SCO can promote adipocyte differentiation. Lipid accumulation assays have been used to screen botanical extracts for effects on adipocyte differentiation (13–16) and to isolate anti-adipogenic compounds from natural products (17, 18), but this is the first study, to our knowledge, to use such screening assays to identify fractions of a botanical extract capable of promoting adipogenesis. Although additional efforts will be required to unequivocally identify pure bioactive compounds from SCO, our study has ruled out several bioactives of SCO and revealed groups of compounds that account for the ability of this plant extract to promote adipocyte development.

## Materials and Methods

### Source and Preparation of *A. scoparia* Extract and Fractions

Ethanolic extracts were prepared from greenhouse-grown plants as described previously (11, 12). The ethanolic extract of *Artemisia scoparia* (SCO, 10 g) was dissolved in water (200 ml) and partitioned 3 times with hexane (3 × 200 ml). Hexane partitions were combined and dried by rotary evaporation to produce the H crude fraction. The water portion was further partitioned 3 times with ethyl acetate (3 × 200 ml). The ethyl acetate partitions were combined and dried by rotary evaporation to produce the EA crude fraction. Remaining water was dried of residual solvents by rotary evaporation and by freeze drying to produce the W crude fraction.

The SCO EA crude fraction was fractionated using a semipreparatory high-performance liquid chromatography (HPLC) system consisting of Waters^TM^ Alliance e2695 Separations Module and 2998 Photodiode Array Detector with a Phenonmenex Synergi 4 μm 80 Å Hydro-RP column 250 × 21.2 mm. The mobile phases consisted of two components: Solvent A (0.5% ACS grade acetic acid in double distilled de-ionized H_2_O), and Solvent B (acetonitrile). Separation was completed using a gradient run of 25% B in A to 95% B over 35 min at a flow rate of 8 mL/min. Five fractions, H1–H5, were obtained.

The SCO EA crude fraction was also subjected to fast centrifugal partition chromatography (FCPC) fractionation using countercurrent chromatography separation (CCS) with a biphasic solvent system (HEMWat +3) composed of Hexanes/ Ethyl-Acetate/ Methanol/ Water (6:4:6:4 v/v) on a Kromaton (Annonay, France) bench-scale fast centrifugal partition chromatography system FCPC1000, v 1.0, equipped with a 1,000 ml volume rotor. For fractionation, the column was first filled with the lower phase 40 ml/min using a Chrom Tech® PR-Pump while rotating at 300 rpm. The system was then equilibrated with upper phase at a flow rate of 10 ml/min and 750 rpm. A 2 g sample of SCO was suspended in 10 ml of each phase of the solvent system used for separation, sonicated and filtered (Millipore filter type NY11). The sample was injected with the upper phase flow at 10 ml/min and rotor rotation of 750 rpm. UV detection was performed at 254 nm using a Visacon VUV-24 detector, and fractions were collected manually. Ten fractions, F1–F10, were obtained.

F2 of the above FCPC fractions was further fractionated using a semipreparatory HPLC system consisting of Waters^TM^ 600 Controller and 486 Tunable Absorbance Detector with a Phenonmenex Synergi 10 μm 80 Å MAX-RP column 250 × 21.2 mm. The mobile phases consisted of two components: Solvent A (0.1% ACS grade acetic acid in double distilled de-ionized H_2_O), and Solvent B (100% acetonitrile). For the initial separation, a gradient run of 40% B in A to 70% B over 35 min was used at a flow rate of 15 ml/min. SCO fraction 2–2 was further fractionated using the same semipreparatory HPLC system with a Phenomenex Kinetex 5 μm C18 100 Å LC column 250 × 10.0 mm. The mobile phases consisted of two components: Solvent A (0.1% ACS grade formic acid in double distilled de-ionized H_2_O), and Solvent B (100% acetonitrile). For the secondary separation, a gradient run of 40% B in A to 80% B over 80 min was used at a flow rate of 5 ml/min. Fractions were collected manually as defined by UV (254 nm) peak designations for both separations.

### UPLC/MS Analysis of *A. scoparia* Extracts

Compounds in samples were separated and analyzed by a UPLC/MS system including the Dionex® UltiMate 3000 RSLC ultra-high pressure liquid chromatography system, consisting of a workstation with ThermoFisher Scientific's Xcalibur v. 4.0 software package combined with Dionex®'s SII LC control software, solvent rack/degasser SRD-3400, pulseless chromatography pump HPG-3400RS, autosampler WPS-3000RS, column compartment TCC-3000RS, and photodiode array detector DAD-3000RS. After the photodiode array detector, the eluent flow was guided to a Q Exactive Plus Orbitrap high-resolution high-mass-accuracy mass spectrometer (MS). Mass detection was full MS scan with low energy collision induced dissociation (CID) from 100 to 1,000 m/z in either positive or negative ionization mode with electrospray ionization (ESI) interface. Sheath gas flow rate was 30 arbitrary units, auxiliary gas flow rate was 7, and sweep gas flow rate was 1. The spray voltage was 3,500 volts (−3,500 for negative ESI) with a capillary temperature of 275°C. The mass resolution was 70,000 or higher. Substances were separated on a Phenomenex^TM^ Kinetex C8 reverse phase column, size 100 × 2 mm, particle size 2.6 μm, pore size 100 Å. The mobile phase consisted of 2 components: Solvent A (0.5% ACS grade acetic acid in LCMS grade water, pH 3–3.5), and Solvent B (100% acetonitrile, LCMS grade). The mobile phase flow was 0.20 ml/min, and a gradient mode was used for all analyses. The initial conditions of the gradient were 95% A and 5% B; for 30 min the proportion reaches 5% A and 95% B which was kept for the next 8 min, and during the following 4 min the ratio was brought to initial conditions. An equilibration interval of 8 min was included between subsequent injections. The average pump pressure using these parameters was typically around 3,900 psi for the initial conditions. Putative formulas of natural products were determined by performing isotope abundance analysis on the high-resolution mass spectral data with Xcalibur v. 4.0 software and reporting the best fitting empirical formula. Database searches were performed using www.reaxys.com (Elsevier RELX Intellectual Properties SA) and SciFinder (scifinder.cas.org, American Chemical Society).

### Cell Culture and Treatments

Murine 3T3-L1 preadipocytes were grown to confluence as described previously (11). Two days post confluence, differentiation was induced using half-strength standard methylxanthine-dexamethasone-insulin (MDI) cocktail (0.25 mM 3-isobutylmethylxanthine (IBMX), 0.5 mM dexamethasone and 0.85 μM insulin) in high-glucose Dulbecco's Modified Eagle's Medium (DMEM) supplemented with 10% fetal bovine serum (FBS). Extracts or fractions dissolved in dimethylsulfoxide (DMSO), or DMSO vehicle only, were added to the differentiation medium. Forty-eight hours after induction of differentiation, medium was replaced with DMEM plus 10% FBS containing 0.425 μM insulin, and cells were again treated with DMSO vehicle or extract/fractions. Cells were either harvested for RNA purification or fixed and stained for lipid accumulation. DMEM, IBMX, dexamethasone, insulin, and DMSO were purchased from Sigma-Aldrich (St. Louis, MO), and FBS from Hyclone (GE Healthcare Life Sciences, Logan, UT).

### RNA Purification and Gene Expression Analysis

Three or four days after induction, RNA was purified from harvested cells using the RNeasy mini-kit (Qiagen, Hilden, Germany) and reverse-transcribed using the High-Capacity cDNA Reverse Transcription kit (Applied Biosystems, Foster City, CA), according to manufacturers' instructions. Quantitative PCR (qPCR) gene expression analyses were performed using primers from Integrated DNA Technologies (Skokie, IL) (primer sequences shown in Table 1) and SYBR Premix (Takara Bio USA, Mountain View, CA). Assays were run on the Applied Biosystems 7900HT system and data were analyzed with SDS 2.3 software. Cycling conditions were as follows: 2 min, 50°C; 10 min, 95°C; 40 cycles of 15 s at 95°C and 1 min at 60°C; dissociation stage: 15 s, 95°C; 15 s, 60°C; 15 s, 95°C. Relative quantities of target genes were normalized to those of the reference gene, non-POU-domain-containing, octamer binding protein (*Nono*).

**Table 1:** qPCR primer sequences for gene expression analyses.

**Gene name and symbol**	**Forward Primer, 5^**′**^-3^**′**^**	**Reverse Primer, 5^**′**^-3^**′**^**
Adiponectin (*Adipoq*)	TGTCTGTACGATTGTCAGTGG	GCAGGATTAAGAGGAACAGGAG
Adipocyte protein 2 (aP2)/Fatty acid binding protein 4 (*Fabp4)*	CCCTCCTGTGCTGCAGCCTTTC	GTGGCAAAGCCCACTCCCACTT
Fatty acid synthase (*Fasn*)	GGCATCATTGGGCACTCCTT	ACCAACAGCTGCCATGGATC
Non-POU-domain-containing, octamer binding protein (*Nono*)	CATCATCAGCATCACCACCA	TCTTCAGGTCAATAGTCAAGCC

### Lipid Accumulation

Four days after the induction of differentiation, cells were fixed with 10% neutral buffered formalin obtained from Thermo Fisher Scientific (Waltham, MA), and stained with Oil Red O (ORO), as described previously (11). Stain was eluted in isopropyl alcohol (IPA), and absorbance measured at 520 nm for quantitation. ORO and IPA were purchased from Sigma-Aldrich.

### Statistics

Statistical analyses were performed using GraphPad Prism software (La Jolla, CA, USA). For control vs. SCO or parent fraction positive controls, *t*-tests were performed with a threshold for statistical significance of *p* < 0.05. Analysis of each test fraction or subfraction was performed using one-way ANOVA with Dunnett's multiple comparisons test (each dose compared to DMSO control).

## Results

**Figure 1:**
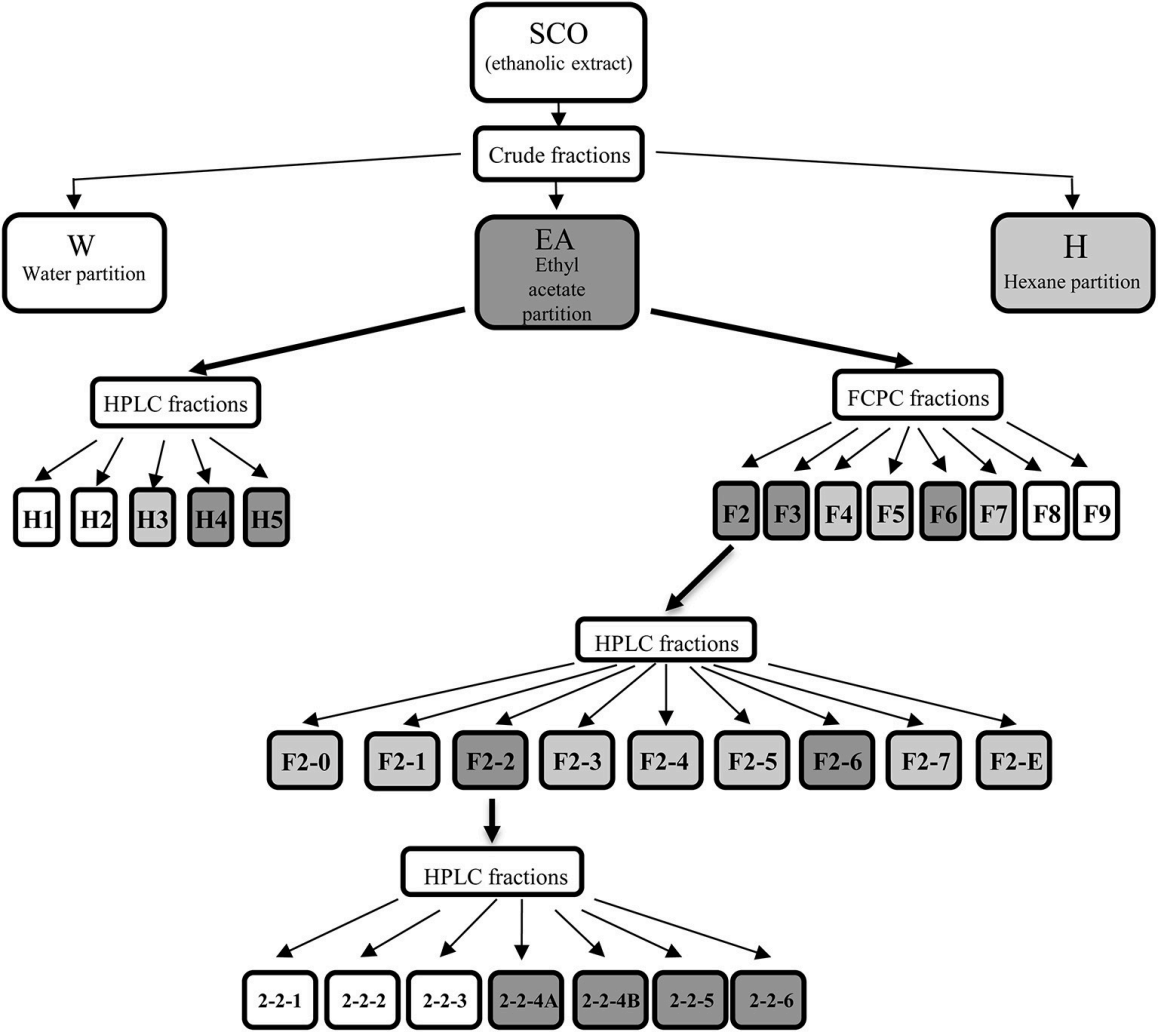
*Artemisia scoparia* extracts, fractions, and subfractions progeny. Shaded fractions and subfractions were active. For crude fractions: light shading indicates significant enhancement of lipid accumulation as measured by ORO staining, while dark shading indicates FC > 1.5 in ORO staining vs. controls. For HPLC fractions of EA, light shading indicates significant effects on ORO staining and adipogenic gene expression at 20 μg/ml dose; dark shading indicates significant effects on ORO staining and gene expression at 5 and 20 μg/ml doses. For FCPC fractions of EA, light shading indicates significant effects on ORO staining at both doses tested (2 and 10 μg/ml); dark shading indicates fractions that enhanced ORO staining by 2-fold or greater. For subfractions of EA-F2, light shading indicates a significant effect on ORO staining at any dose and FC < 1.5, while dark shading indicates fractions that enhanced ORO staining by 1.5-fold or more at any dose. For subfractions of F2-2, shading indicates significant increases in ORO staining and in *AdipoQ* and *Fabp4* gene expression at both doses tested.

We have previously shown that SCO exerts robust pro-adipogenic effects in the 3T3-L1 preadipocyte cell line and that SCO can activate the PPARγ ligand binding domain in HEK 293 cells when examined using a Gal4 DNA-binding-luciferase assay (10, 11). In addition to these *in vitro* studies, we have also demonstrated that SCO has some protective effects against diet-induced obesity in mice (10, 11). We predict that the metabolically beneficial effects of SCO *in vivo* might be mediated by its ability to increase adipogenesis. In order to identify bioactive components of SCO, crude partitions of SCO (water, hexane, and ethyl acetate) and their subfractions were tested for effects on adipogenesis in 3T3-L1 cells. An outline diagram of the fractionation scheme is shown in Figure 1.

**Figure 2:**
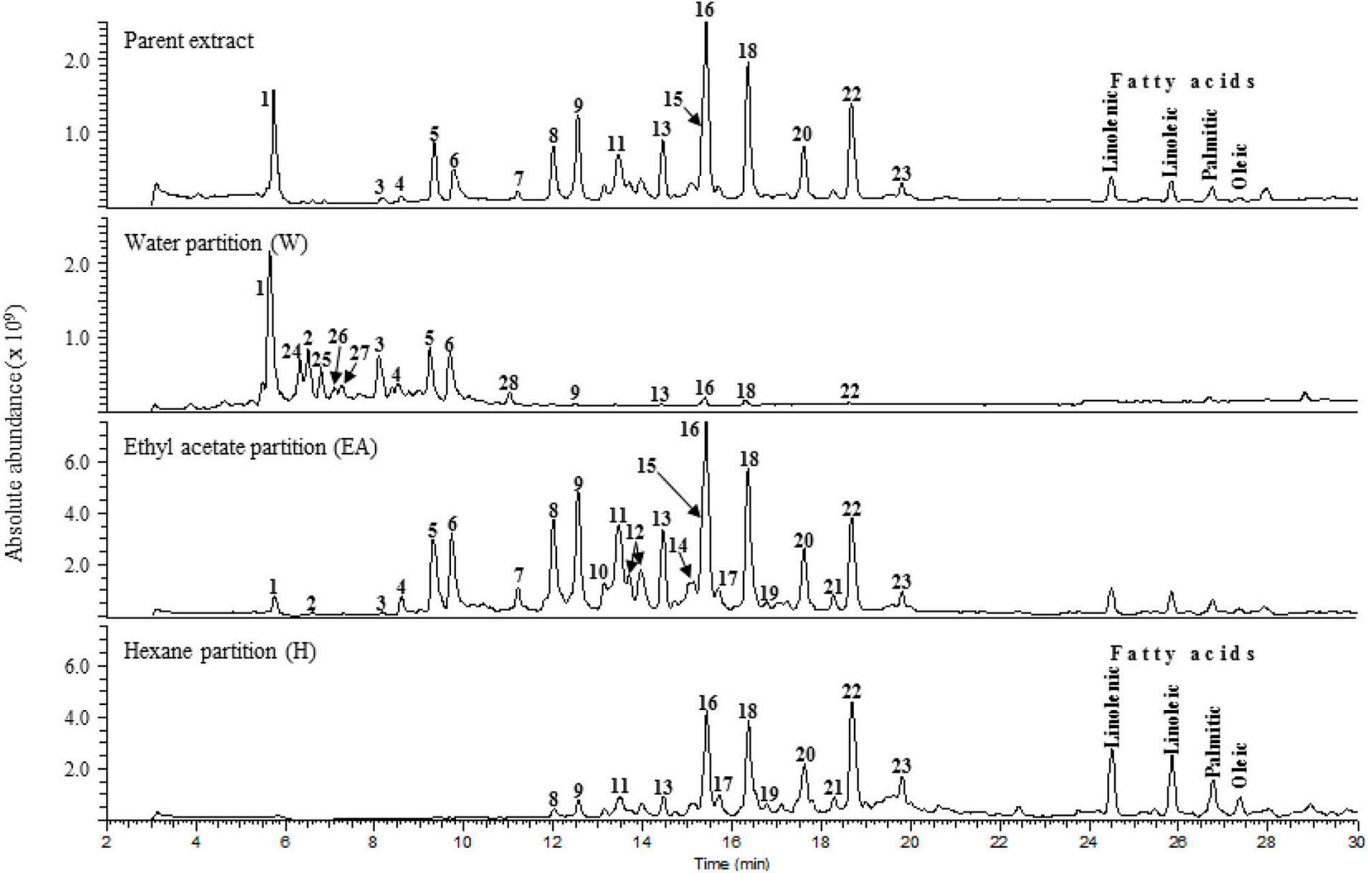
Total ion current [(-)ESI MS] chromatograms of SCO (Parent extract) and its water, ethyl acetate, and hexane fractions.

LC-MS analysis was performed on the parent SCO extract and the three crude partitions (Figure 2). Twenty-eight constituent compounds were detected, and putatively identified (Table 2). The ethyl acetate fraction (EA) was found to have an enriched profile relative to that of the parent SCO extract, and all 23 compounds detected in SCO were also present in the EA fraction. The hexane (H) and water (W) fractions contained non-overlapping subsets of peaks (peaks 1–6 predominant in W, and peaks 16–23 in H). W also contained 5 additional peaks (peaks 24–28) that were not detected in SCO, indicating a low concentration of the corresponding compounds in the parent extract.

**Table 2:** Preferred molecular formulas and putative identification of compounds detected by UPLC/MS in SCO: Preferred molecular formulas were determined from isotope abundance analysis (Xcalibur v. 4.0 software), and compounds were putatively identified through searches of natural product databases.

**Peak #**	**[M – H] (Preferred mol. formula)**	**Reported from *Artemisia* species (Reaxys.com)**	**Reported as natural product (Reaxys.com)**
1	353.0873 (C_16_H_18_O_9_)	Chlorogenic acid	
2	311.0769 (C_14_H_16_O_8_)	No	Caffeic acid glycosides
3	609.1457 (C_27_H_30_O_16_)	Rutin	
4	463.0875 (C_21_H_20_O_12_)	Quercetin-glycoside	
5	515.1181 (C_25_H_24_O_12_)	Di-caffeoyl-quinic acid	
6	515.1181 (C_25_H_24_O_12_)	Di-caffeoyl-quinic acid	
7, 8, 9	331.1547/331.1548 (C_19_H_24_O_5_)	Prenylated cinnamic acid(s) and/or Sesquiterpene lactone(s)	
10	303.1599 (C_18_H_24_O_4_)	Prenylated acetophenone	
11	285.0399 (C_15_H_10_O_6_)	6-Demethoxycapillarisin	
12	359.0772 (C_18_H_16_O_8_)	Trihydroxy-tri-MeO-flavones	
13	373.1653 (C_21_H_26_O_6_)	A coumarin monoterpene ether and/or a lignan	
14	343.0823 (C_18_H_16_O_7_)	A Dihydroxy-tri-MeO-flavone	
15	373.1654 (C_21_H_26_O_6_)	A coumarin monoterpene ether and/or a lignan	
16	315.1601 (C_19_H_24_O_4_)	Prenylated coumaric acid or Artepillin A	
17	315.1601 (C_19_H_24_O_4_)	Prenylated coumaric acid or Artepillin A	
18	315.1601 (C_19_H_24_O_4_)	Prenylated coumaric acid or Artepillin A	
19	373.1654 (C_21_H_26_O_6_)	A coumarin monoterpene ether	
20	415.1759 (C_23_H_28_O_7_)	(Epi-)Magnolin	Schisandrin type of structures; Lignan derivatives
21	357.1713 (C_21_H_26_O_5_)	Prenylated coumaric acid	
22	357.1713 (C_21_H_26_O_5_)	Prenylated coumaric acid	
23	299.1649 (C_19_H_24_O_3_)	Artepillin C or a steroid	
24	387.1662 (C_18_H_28_O_9_)	No	A hydroxyphenyl monosaccharide
25	755.2044 (C_33_H_40_O_20_)	No	Quercetin trisaccharide
26	471.1148 (C_20_H_24_O_13_)	No	A coumarin disaccharide
27	695.1478 (C_30_H_32_O_19_)	No	A flavone malonyldisaccharide
28	427.1611 (C_20_H_28_O_10_)	No	A hydroxybenzoyl disaccharide

**Figure 3:**
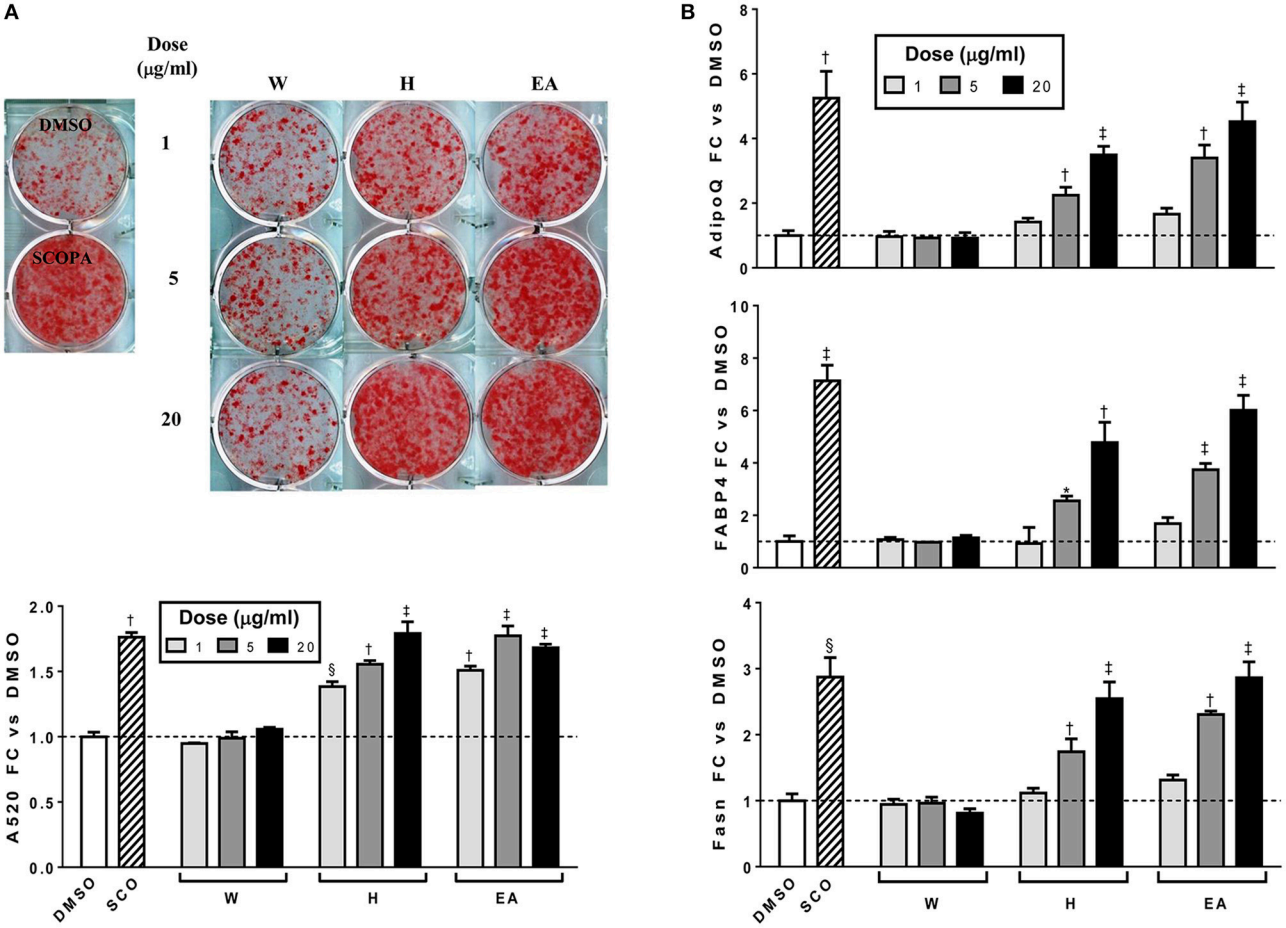
Hexane (H) and ethyl acetate (EA) fractions of SCO enhance adipogenesis in 3T3-L1 cells. Cells were induced to differentiate using half-strength MDI cocktail containing DMSO vehicle, 50 μg/ml of SCO, or 1, 5, or 20 μg/ml of W, H, or EA. Four days after induction, cells were fixed and stained with Oil Red O. Staining was quantitated by measuring absorbance in isopropanol eluates **(A)**. Three days after induction, cells were harvested, RNA was isolated and subjected to reverse transcription and qPCR to assess expression of the adipogenic genes *AdipoQ, Fabp4*, and *Fasn* **(B)**. All data are expressed as fold-change vs. vehicle controls. ^*^p < 0.05, ^§^*p* < 0.01, ^†^*p* < 0.001, ^‡^*p* < 0.0001 vs. DMSO controls. Results have been replicated in two additional experiments on independent batches of cells.

In order to determine which compound(s) or combination(s) of compounds could enhance adipogenesis, we tested the crude partitions for effects on lipid accumulation and on the expression of several genes known to be induced during adipocyte development. These genes include the adipocyte specific hormone adiponectin (*AdipoQ*), fatty acid binding protein 4/adipocyte protein 2 (*Fabp4*), and fatty acid synthase (*Fasn*). Results are shown in Figure 3A (lipid accumulation) and Figure 3B (adipogenic gene expression). Lipid accumulation, as measured by ORO staining, was not affected by treatment with 1, 5, or 20 μg/ml of W. Fractions H and EA both significantly enhanced lipid accumulation at all three doses tested, with the 20 μg/ml dose of H and the 5 and 20 μg/ml doses of EA showing effects similar to that of the SCO parent extract. Adipogenic gene expression was consistent with the lipid accumulation results, with no effect of W on the expression of *AdipoQ, Fabp4, or Fasn*, and clear dose-dependent increases in all three genes with H and EA. However, the effects of H and EA at the low dose (1 μg/ml) were not significant. The active hexane fraction (H), whose most abundant components were peaks 16, 18, 20, and 22, was only slightly less active than the ethyl acetate fraction (EA), which contained all 23 of the peaks identified in SCO. The inactive water fraction (W) contained mainly chlorogenic acid (peak 1) and caffeic acid and quercetin derivatives, including two dicaffeoylquinic acids (peaks 2–6) (LC-MS data shown in Figure 2).

**Figure 4:**
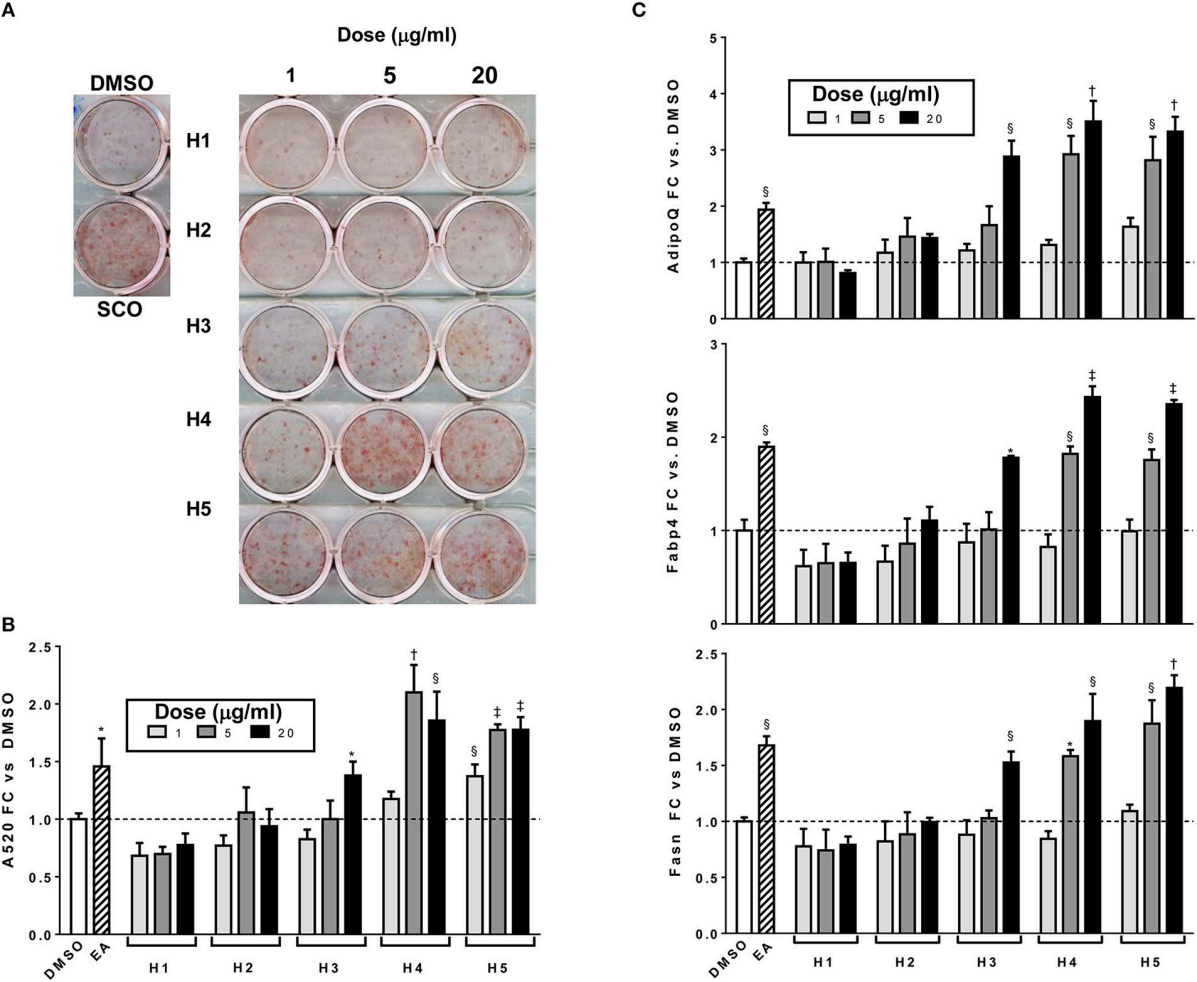
Adipogenesis in 3T3-L1 cells is enhanced by HPLC subfractions of EA. Cells were induced to differentiate using half-strength MDI cocktail containing DMSO vehicle, 20 μg/ml of EA, or 1, 5, or 20 μg/ml of each HPLC subfraction of EA. Four days after induction, cells were fixed and stained with Oil Red O **(A)**; absorbance was measured in isopropanol eluates to quantitate staining **(B)**. Cells were also harvested for RNA isolation and reverse transcription, expression of adiponectin (*AdipoQ*), aP2 (*Fabp4*), and FAS (*Fasn)* were assayed by qPCR **(C)**. All data expressed as fold-change vs. DMSO vehicle controls. ^*^p < 0.05,^§^ *p* < 0.01, ^†^*p* < 0.001, ^‡^*p* < 0.0001 vs. DMSO controls.

**Figure 5:**
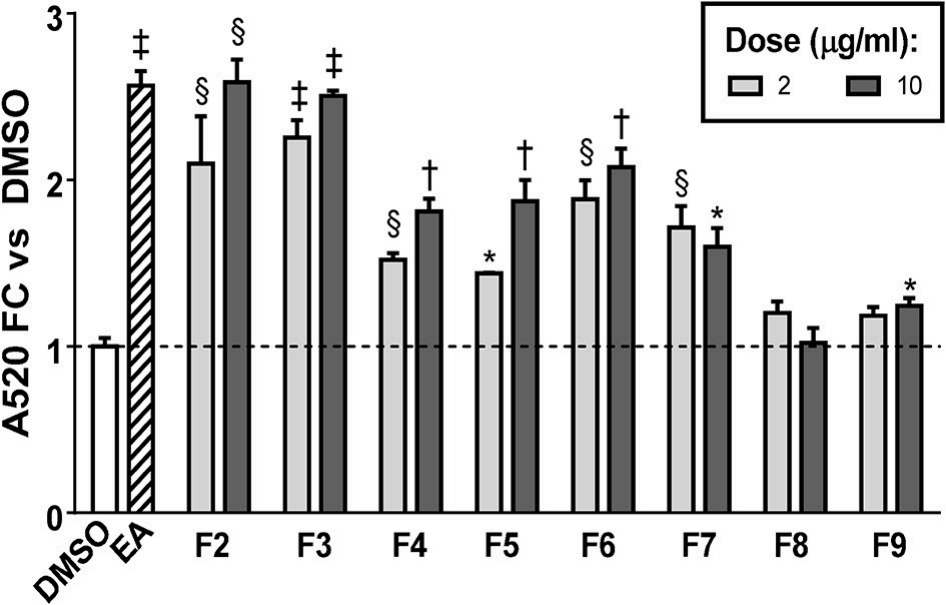
Adipogenesis in 3T3-L1 cells is enhanced by FCPC subfractions of EA. Cells were induced to differentiate using half-strength MDI cocktail containing DMSO vehicle, 20 μg/ml of EA, or 2 or 10 μg/ml of each FCPC subfraction of EA. Four days after induction, cells were fixed and stained with Oil Red O. Staining was quantitated by measuring absorbance in isopropanol eluates. All data expressed as fold-change vs. DMSO vehicle controls. ^*^p < 0.05, ^§^ *p* < 0.01, ^†^*p* < 0.001, ^‡^*p* < 0.0001 vs. DMSO controls. Images of the stained plates are shown in Figure S2. The same fractions were found to be active in two independent experiments.

To further characterize the bioactivity of SCO, two sets of fractions were prepared from EA: one by HPLC and another by FCPC (Figure 1). LC-MS analysis and putative compound identification for the most active HPLC and FCPC fractions are shown in Figure S1. Of the five HPLC fractions tested, H4 and H5 produced a robust induction in lipid accumulation and adipogenic gene expression, H3 produced more modest positive effects, and H1 and H2 were inactive (Figure 4). In the lipid accumulation assay (Figures 4A,B), 5 and 20 μg/ml doses of H4 and H5 enhanced staining even more than their parent EA fraction. At the lowest dose (1 μg/ml) H5 produced a more modest increase in ORO staining, and the effect of H4 was not significant. H1 and H2 had no significant effects on lipid accumulation at any of the doses tested. Effects of the HPLC fractions on adipogenic gene expression closely mirrored the ORO results, with the 5 and 20 μg/ml doses of H4 and H5, as well as the 20 μg/ml dose of H3 strongly inducing the expression of *AdipoQ, Fabp4, and Fasn*, and H1 and H2 having no significant effects on any of the genes (Figure 4C). H3 consisted almost exclusively of peaks 7, 8, and 9 (all identified as prenylated cinnamic acids and/or sesquiterpene lactones with a m/z of 331 [M-H]^+^). Peaks identified as prenylated coumaric acid or Artepillin A (m/z of 315 [M-H]^+^) were the most abundant in both H4 and H5. H4 also contained relatively large amounts of peaks 11, 12, 13, and 15, whereas H5 had significant amounts of peaks 20 and 22, as well as an additional compound putatively identified as a prenylated acetophenone. In the inactive fractions (H1 and H2), we observed the presence of peaks #1–6 (chlorogenic acid, caffeic acid glycosides, rutin, quercetin glycoside and dicaffeoylquinic acids). These results are consistent with those obtained from our analysis of the crude fractions (EA, H, and W).

FCPC was used as an alternative method of fractionation to HPLC because larger amounts of extract could be separated in each run. Quantitation of ORO staining for the FCPC fractions of EA is shown in Figure 5 (images of stained plates are shown in Figure S2). The most active FCPC fractions of EA were F2, F3, and F6. F2 and F3 enhanced lipid accumulation by at least two-fold at both doses tested (2 and 10 μg/ml), while fold-changes for F6 were slightly lower; F4, F5, and F7 significantly increased ORO staining, but to a lesser degree than F2, F3, or F6. F8 and F9 were inactive (with the exception of very slight enhancement of lipid accumulation at the higher dose of F9). Once again, peaks 1 through 6 were the major constituents of the inactive fractions (F8 and F9), while the active fractions (F2 through F7) contained combinations of peaks 8, 9, 11–13, 15, 16, 18, 20, and 22.

**Figure 6:**
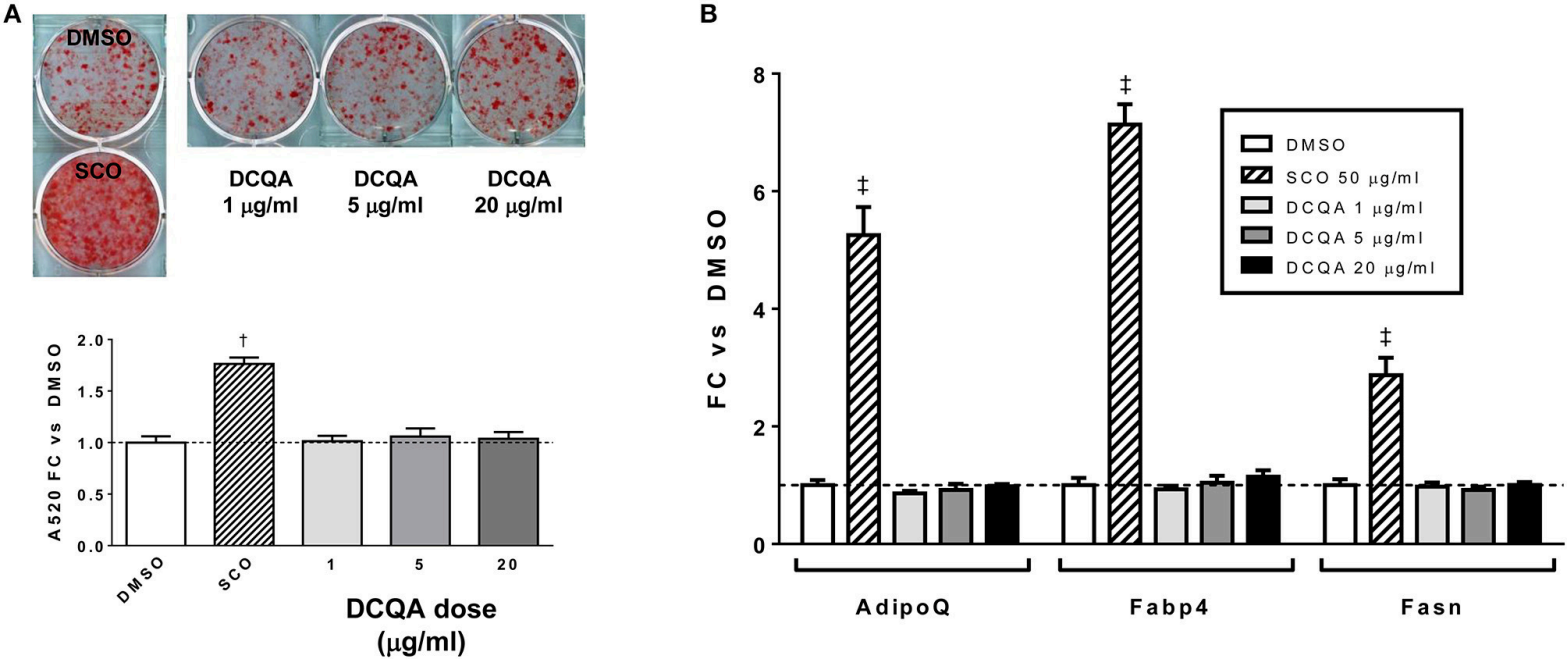
3,5-DCQA does not enhance adipogenesis in 3T3-L1 cells. Cells were induced to differentiate using half-strength MDI cocktail containing DMSO vehicle, 50 μg/ml SCO, or 1, 5, or 20 μg/ml of 3, 5-dicaffeoylquinic acid (DCQA). **(A)** 4 days after induction, cells were fixed and stained with Oil Red O. Staining was quantitated by measuring absorbance in isopropanol eluates. **(B)** 3 days after induction, cells were harvested for RNA purification and reverse transcription; relative expression of *AdipoQ, Fabp4*, and *Fasn* were assayed by qPCR. All data expressed as fold-change vs. DMSO vehicle controls. ^†^*p* < 0.001, ^‡^*p* < 0.0001 vs. DMSO controls. Three independent experiments were performed with the same results.

To summarize, adipogenesis experiments with SCO crude partitions (EA, H, and W) and with HPLC or FCPC fractions of EA provided highly consistent results demonstrating that the fractions containing predominantly prenylated coumaric acid derivatives, prenylated cinnamic acids and/or sesquiterpene lactones, 6-demethoxycapillarisin, polymethoxyflavones, and coumarin monoterpene ethers promoted adipogenesis in 3T3-L1 cells, while fractions containing chlorogenates and quercetin derivatives did not. A compound detected in our inactive fractions, 3,5-dicaffeoylquinic acid (peak 5), has previously been isolated from *A. scoparia* and shown to have anti-inflammatory effects in activated mast cells (19). To determine whether it could regulate adipogenesis in our experimental conditions, we treated differentiating 3T3-L1 cells with commercially obtained 3,5-DCQA and did not observe any effects on lipid accumulation or adipogenic gene expression (Figure 6). While there is substantial evidence that chlorogenic acid (CGA, peak 1) has beneficial effects on metabolic parameters *in vivo* (20), pro- and anti-adipogenic, as well as neutral effects on adipocyte differentiation have been reported for CGA and for botanical extracts containing CGA (21–27). In our hands, CGA had no effect on lipid accumulation during 3T3-L1 differentiation (data not shown), consistent with our findings that the SCO fractions containing CGA did not enhance adipogenesis. Previous SCO fractionation efforts in our laboratory have yielded several pure compounds corresponding to peaks in the W fraction, and these also failed to promote adipocyte differentiation.

**Figure 7:**
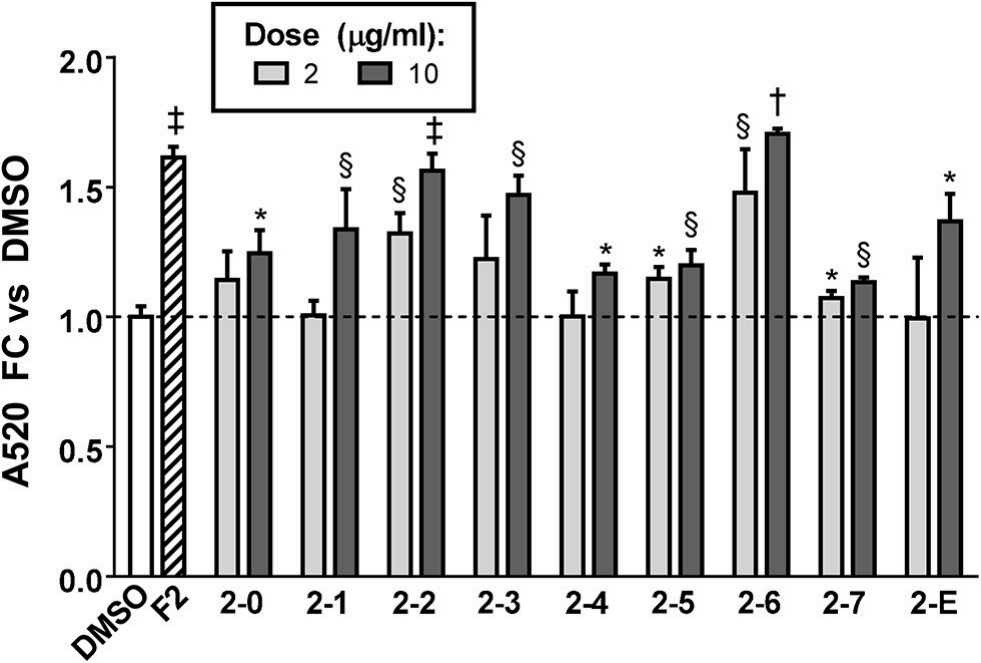
Adipogenesis in 3T3-L1 cells is enhanced by subfractions of EA-F2. Cells were induced to differentiate using half-strength MDI cocktail containing DMSO vehicle, 10 μg/ml of EA-F2, or 2 or 10 μg/ml of each EA-F2 subfraction. 4 days after induction, cells were fixed and stained with Oil Red O. Staining was quantitated by measuring absorbance in isopropanol eluates. All data expressed as fold-change vs. DMSO vehicle controls. ^*^p < 0.05, ^§^*p* < 0.01, ^†^*p* < 0.001, ^†^*p* < 0.0001 vs. DMSO controls. Images of the stained plates are shown in Figure S3.

Having conclusively determined the lack of activity of SCO fractions containing chlorogenates and quercetin derivatives, we proceeded to subfractionate F2, as this fraction contained relatively large amounts of peaks 16, 18, 20, and 22. Subfractions F2-0 through F2-7, plus F2-E were tested for effects on lipid accumulation in differentiating 3T3-L1 cells (LC-MS data for F2 shown in Figure S1). Quantitation of lipid staining by ORO is shown in Figure 7 (Figure S3 contains images of stained plates). All subfractions of F2 enhanced lipid accumulation to some degree, with F2-2, F2-3, and F2-6 showing the most activity. As was the case with the HPLC fractions of EA, we observed that fractions with little or no overlap were capable of enhancing adipogenesis. Indeed, LC-MS analysis determined that F2-2 was comprised of peaks 15, 16, and 17. While F2-3 and F2-6, both very active in promoting adipogenesis, were not pure single compounds, they each had a single very dominant peak. In the case of F2-3, that peak was peak 17 (putatively identified as prenylated coumaric acid or Artepillin A), while the principal constituent of F2-6 was peak 22 (another prenylated coumaric acid).

**Figure 8:**
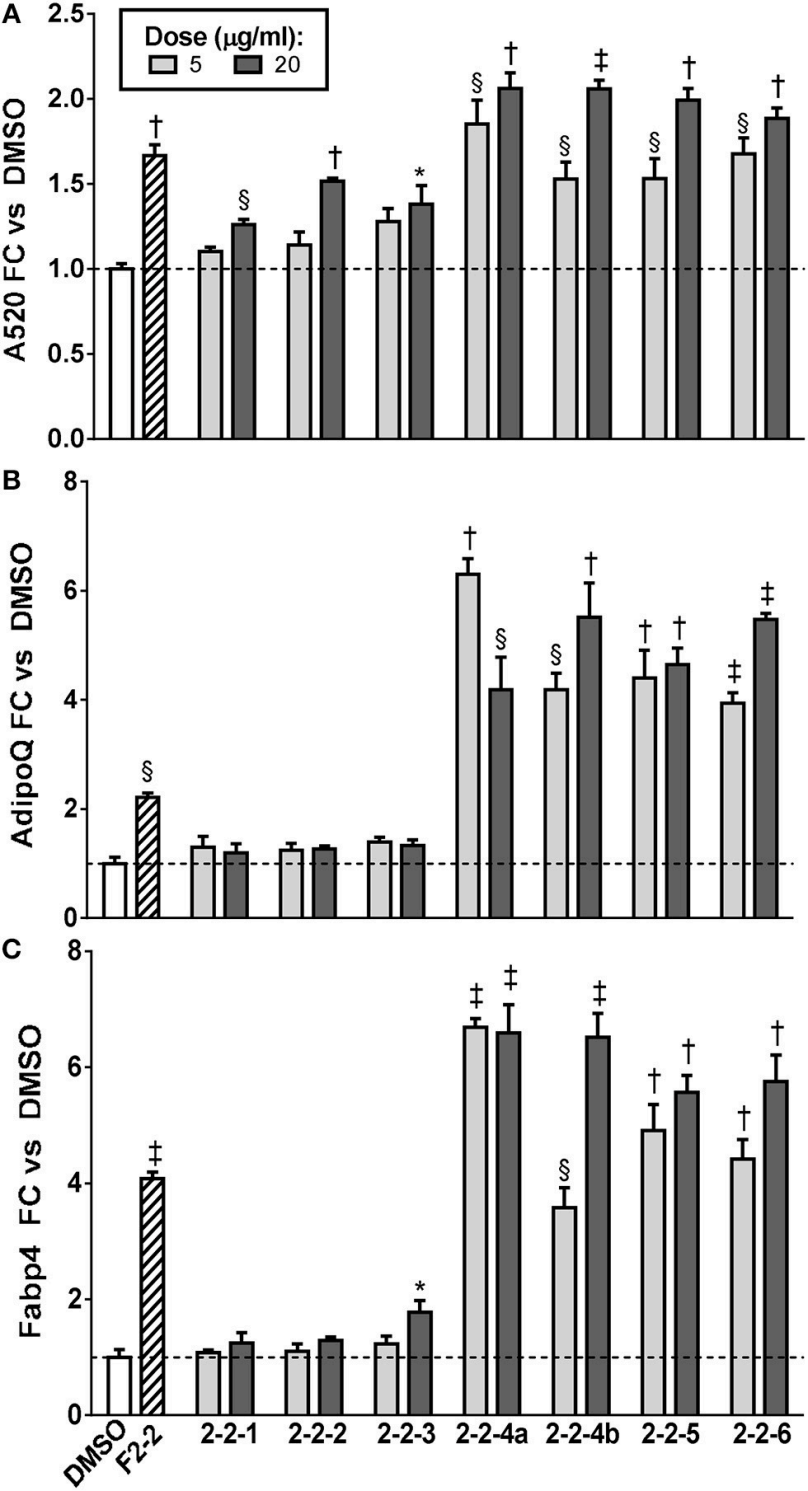
Adipogenesis in 3T3-L1 cells is enhanced by subfractions of EA-F2-2. Cells were induced to differentiate using half-strength MDI cocktail containing DMSO vehicle, 10 μg/ml of F2-2, or 5 or 20 μg/ml of each EA-F2 subfraction. **(A)** 4 days after induction, cells were fixed and stained with Oil Red O. Staining was quantitated by measuring absorbance in isopropanol eluates. **(B)** 3 days after induction, cells were harvested for RNA purification and reverse transcription; relative expression of *AdipoQ* and *Fabp4* were assayed by qPCR. All data expressed as fold-change vs. DMSO vehicle controls. ^*^p < 0.05, ^§^*p* < 0.01,^†^*p* < 0.001, ^‡^*p* < 0.0001 vs. DMSO controls. Images of the stained plates are shown in Figure S4.

Since F2-2 contained a higher number of peaks than F2-3 or F2-6, we further fractionated F2-2 into 7 subfractions (2-2-1, 2-2-2, 2-2-3, 2-2-4A, 2-2-4B, 2-2-5, and 2-2-6) that were examined for their potential ability to modulate adipogenesis. Quantitation of ORO staining for F2-2 subfractions is shown in Figure 8 and images of the stained plates are in Figure S4. F2-2-1, 2-2-2, and 2-2-3 slightly enhanced lipid accumulation at the highest dose tested, but did not increase expression of *AdipoQ or Fabp4*. In contrast, F2-2-4a, 2-2-4b, 2-2-5, and 2-2-6 robustly promoted lipid accumulation and adipogenic gene expression at both doses tested. Although the four main constituents we had identified in F2-2 were not further resolved by this subsequent fractionation, they did, as a group, segregate into the active subfractions (F2-2-4a, 2-2-4b, 2-2-5, and 2-2-6). The inactive subfractions, in contrast, contained peaks that were much less abundant in the parent F2-2 fraction. These observations provide further evidence that the combination of F2-2's dominant compounds promotes adipogenesis. Interestingly, although peak 9 (prenylated cinnamic acids and/or SQLs) was present only in very small amounts in the parent F2-2 fraction, it was a major constituent of F2-2's inactive fractions. Indeed, peak 9 and additional higher-retention-time (RT) peaks also identified as C_19_H_24_O_5_ were found to be the predominant constituents of F2-2-1, F2-2-2, and F2-2-3 (data not shown), indicating that these peaks are not responsible for the pro-adipogenic effects of SCO or its active fractions.

## Discussion

As the current obesity epidemic continues unabated, the need for novel interventions to enhance metabolic health remains significant. Adipose tissue is an attractive target for preventive and therapeutic strategies that should include botanical bioactives and their derivatives. Our group has identified an extract of *Artemisia scoparia* (SCO) as an enhancer of adipocyte differentiation and function *in vitro*, and of whole-body insulin sensitivity *in vivo* (10, 11). To better understand the mechanisms involved in SCO's effects on adipogenesis and to identify individual compounds in SCO that may be responsible for those effects, we performed activity-guided fractionation, in which fractions and subfractions were tested for bioactivity in differentiating 3T3-L1 adipocytes, and analyzed by LC-MS for composition. The ability of SCO to enhance adipogenesis was measured by examining both lipid accumulation and the expression of genes induced during adipocyte development.

Crude partitioning of SCO yielded two fractions with pro-adipogenic activity (H and EA), and one inactive fraction (W). Some of the major constituent compounds in the inactive water partition (W) were the lower retention time peaks (higher polarity) found in the SCO parent extract, putatively identified as chlorogenates and quercetin derivatives, including dicaffeoylquinic acids. These classes of compounds have been found to improve insulin sensitivity and metabolic function, but they appear to do so while also inhibiting adipogenesis (24, 26, 28–38). We observed no enhancement or inhibition of adipogenesis in cells treated with the W fraction or with the pure compounds CGA and 3,5-DCQA.

The hexane partition (H), in contrast, was quite active, and contained mostly the higher retention time peaks (non-polar) of the SCO parent extract. The four most abundant peaks were putatively identified as three prenylated coumaric acid peaks, plus one peak tentatively identified as a lignan derivative. The preferred formula of this peak based on high resolution, high mass accuracy mass spectral analysis was C_23_H_28_O_7_. This is the formula for the lignan magnolin, among other natural products, which has been reported in *Artemisia* species; however, analysis of a commercially obtained standard revealed that magnolin standard had a different retention time than the peak in our fractions, so we ruled out magnolin as a bioactive in our SCO fractions. The H fraction was also predictably enriched in four fatty acids (linolenic, linoleic, palmitic, and oleic). Although linoleic and linolenic acids are known to be agonists of PPARγ (16, 39, 40), our data from subsequent subfractions do not support a major role for these compounds in the adipogenic activity of SCO. In addition, unpublished experiments from our laboratory showed no effect of linoleic acid on 3T3-L1 adipocyte differentiation.

Given that EA contained all 23 peaks found in SCO, we opted to subfractionate this partition for further testing, and confirmed that chlorogenates and quercetin derivatives segregated into subfractions that were ineffective in enhancing 3T3-L1 differentiation. However, subfractions rich in other compounds (prenylated coumaric and cinnamic acids, coumarin monoterpene ethers, 6-demethoxycapillarisin, polymethoxyflavones, and possibly sesquiterpene lactones) displayed pro-adipogenic activity. We also observed several active fractions with no overlap in chemical composition, indicating the presence of several individual compounds in SCO capable of improving adipocyte differentiation. The identification of prenylated coumaric acids (PCAs) as putative adipogenic agents in SCO was particularly interesting given that the PCAs drupanin, baccharin, and artepillin C have been identified as major bioactive constituents of propolis and that propolis can enhance differentiation of 3T3-L1 cells (41, 42). In particular, Artepillin C, which is found both in Brazilian green propolis and in the plant from which it is derived, *Baccharis dracunculifolia*, has been shown to promote adipogenesis through activation of PPARγ (43). Although the formulas of SCO compounds thought to be PCAs in our extracts and fractions are different from known adipogenic PCAs, it is certainly possible that SCO contains distinct PCAs with similar properties. As for the other compounds putatively identified in SCO's adipogenic fractions, little is known about their effects on adipogenesis. Polymethoxyflavones are a large group of phytochemicals with a wide range of bioactivity. However, the reported effects of polymethoxyflavones on adipocyte differentiation and insulin resistance are not consistent (44–49). Sesquiterpene lactones, identified as possible constituents of several pro-adipogenic SCO fractions (H3, H4, F3, F5, F6, F7), have been reported to have both pro- and anti-adipogenic activities for different compounds in this class (50–55). In mature 3T3-L1 adipocytes, the coumarin monoterpene auraptene, isolated from citrus, increases adiponectin levels and the proportion of high-molecular-weight adiponectin, and also reduces levels of the pro-inflammatory cytokine monocyte chemoattractant protein 1 (MCP-1) (56). To our knowledge, nothing is known about the effects of this class of compounds on adipocyte differentiation.

The data presented here highlight some of the challenges of investigating the bioactivity of botanical extracts. Identifying individual compounds in such complex mixtures involves methods that are generally time-consuming and expensive. Definitive identification of the constituents of our active SCO fractions will require large amounts of highly purified individual compounds for chemical analysis using nuclear magnetic resonance (NMR) spectroscopy to characterize the molecular structures. As we obtain these pure single compounds, we will test them individually and in combination to assess their ability to enhance adipocyte development, both *in vitro* and *in vivo*. Nevertheless, our novel observations have conclusively determined that compounds related to quercetin and chlorogenic acid do not mediate SCO's adipogenic effects. In addition to enhancing adipogenesis, SCO can exert beneficial effects on endocrine function, inflammation (10, 11), and lipolysis (12) in mature adipocytes; the activity-guided fractionation approach used in this study can be applied to determine the components of SCO which contribute to each of these effects.

In conclusion, our activity-guided fractionation efforts have revealed the presence of multiple pro- and non-adipogenic compounds within *A. scoparia*. While SCO contains several chlorogenic acid and quercetin derivatives with reported bioactivity, these compounds are not responsible for the extract's pro-adipogenic properties. Active fractions were enriched in prenylated cinnamic acid derivatives, coumarin monoterpene ethers, polymethoxyflavones, 6-demethoxycapillarisin, a possible lignan, and perhaps sesquiterpene lactones. The peaks identified as prenylated coumaric acids are of particular interest, since other PCAs have been shown to have effects consistent with enhancing adipogenesis. In addition, non-overlapping fractions were found to be active in our adipogenesis assay, indicating the presence of more than one active compound in SCO. Although the compounds we have putatively identified in the pro-adipogenic fractions of SCO are chemically related to other bioactives shown to have pro-adipogenic or metabolically beneficial effects, they are not identical and, therefore, it appears that SCO contains compounds not previously known to have these effects. The identification of bioactive classes of compounds within SCO sets the groundwork for the characterization of single bioactive compounds and the study of their mechanism(s) of action.

## Author Contributions

JS, AP, and DR conceived and designed experiments. DR, AP, and TR prepared and analyzed the SCO extract and fractions. AB and AP performed experiments and analyzed data. AB, AR, and JS interpreted results. AB and AP prepared figures. AB drafted the manuscript. AB, AR, AP, DR, IR, and JS edited and revised the manuscript. All authors approved the final version of the manuscript.

### Conflict of Interest Statement

The authors declare that the research was conducted in the absence of any commercial or financial relationships that could be construed as a potential conflict of interest.
