# Supplementary material for: Distinct Fractions of an Artemisia scoparia Extract Contain Compounds With Novel Adipogenic Bioactivity
Source: Front Nutr. 2019 Mar 8;6:18. doi: 10.3389/fnut.2019.00018 (PMC6418310; doi:10.3389/fnut.2019.00018)
Supplement: Supplementary file 1 [file Data_Sheet_1.pdf]

Supplementary Material

Distinct fractions of an Artemisia scoparia extract contain compounds with novel adipogenic bioactivity.

Anik Boudreau[1], Alexander Poulev[3], David M. Ribnicky[3], Ilya Raskin[3], Thirumurugan Rathinasabapathy[3], Allison J. Richard[1], and Jacqueline M. Stephens[1,2*]

*** Correspondence:**

Jacqueline Stephens

Louisiana State University

Pennington Biomedical Research Center

6400 Perkins Road

Baton Rouge, LA 70808

Phone (225)-763-2648

FAX (225)-578-2597

jsteph1@lsu.edu

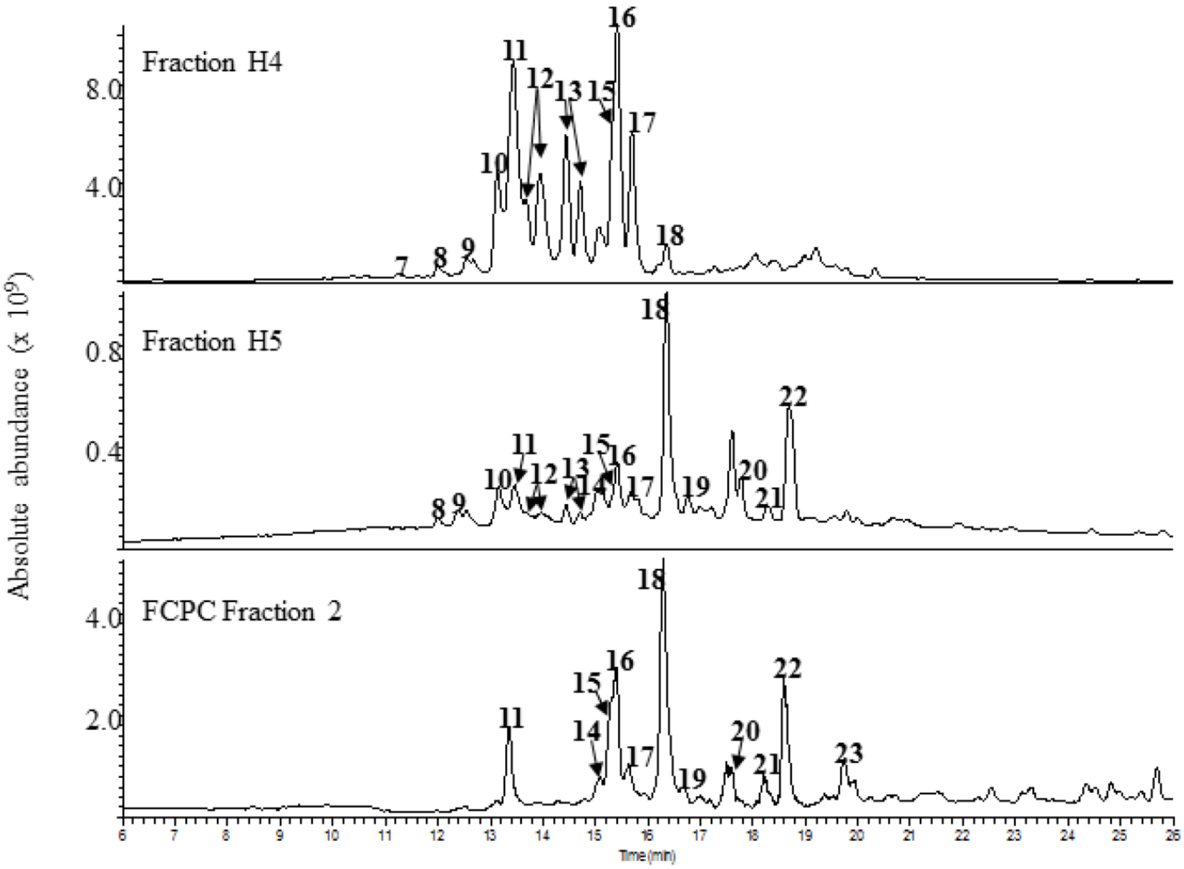

Supplementary Figure 1. (-)ESI MS total ion current chromatograms of three fractions of EA with pro-adipogenic activity. Peak identification is shown in table 2.

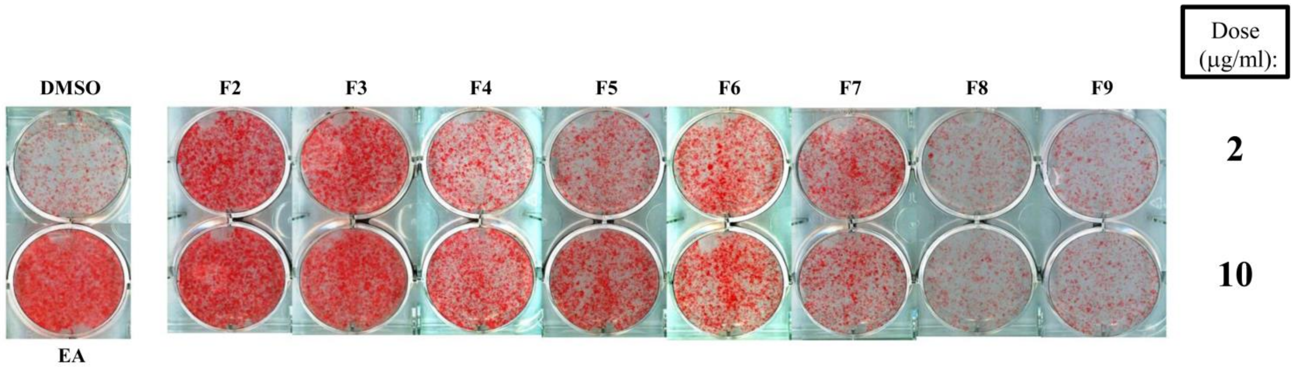

Figure S2. Adipogenesis in 3T3-L1 cells is enhanced by FCPC subfractions of EA. Cells were induced to differentiate using half-strength MDI cocktail containing DMSO vehicle, 20 µg/ml of EA, or 2 or 10 µg/ml of each FCPC subfraction of EA. 4 days after induction, cells were fixed, stained with Oil Red O, and scanned. Wells were treated and stained in triplicate; images shown are from one representative well for each condition.

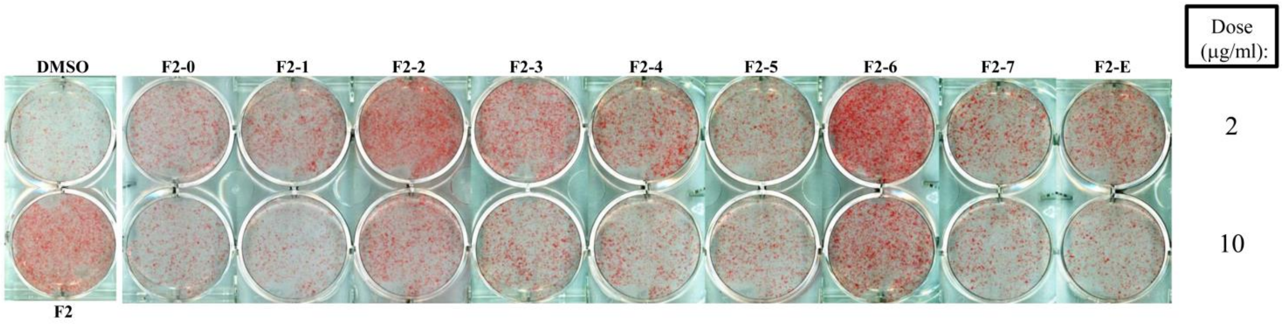

Figure S3. Adipogenesis in 3T3-L1 cells is enhanced by subfractions of EA-F2. Cells were induced to differentiate using half-strength MDI cocktail containing DMSO vehicle, 10 μg/ml of F2, or 2 or 10 μg/ml of each EA-F2 subfraction. 4 days after induction, cells were fixed, stained with Oil Red O, and scanned. Wells were treated and stained in triplicate; images shown are from one representative well for each condition.

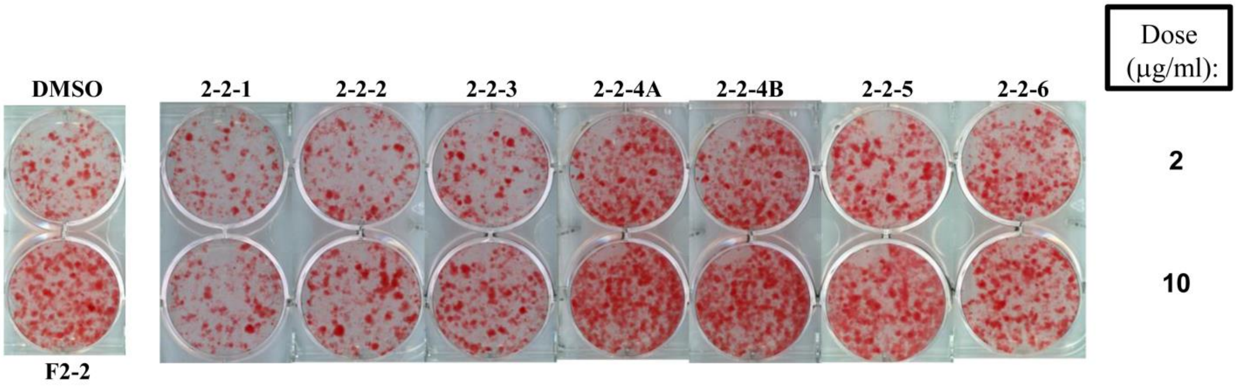

Figure S4. Adipogenesis in 3T3-L1 cells is enhanced by subfractions of EA-F2-2. Cells were induced to differentiate using half-strength MDI cocktail containing DMSO vehicle, 10 μg/ml of F2-2, or 5 or 20 μg/ml of each EA-F2 subfraction. 4 days after induction, cells were fixed, stained with Oil Red O, and scanned. Wells were treated and stained in triplicate; images shown are from one representative well for each condition.
